# Supplementary material for: Candida albicans Hexokinase 2 Challenges the Saccharomyces cerevisiae Moonlight Protein Model
Source: Microorganisms. 2021 Apr 15;9(4):848. doi: 10.3390/microorganisms9040848 (PMC8071269; doi:10.3390/microorganisms9040848)
Supplement: Supplementary file 1 [file microorganisms-09-00848-s001.zip › microorganisms-1161426-SI.pdf]

Supplementary data

Table S1. *C. albicans* strains used in this study

Strains	Parent	Genotype	Source/reference
WT (SC5314)		Wild-type strain	Gillium et al., 1984
Cahxk2Δ/Δ	SC5314	*Δhxk2::FRT/ Δhxk2::FRT*	Laurian et al., 2019
Cahxk2D^{210}A	*Cahxk2Δ/Δ*	*Cahxk2D^{210}A::FRT/ Cahxk2D^{210}A::FRT*	This study
Cahxk2T^{15}A	*Cahxk2Δ/Δ*	*Cahxk2T^{15}A::FRT/ Cahxk2T^{15}A::FRT*	This study
Cahxk2ΔK^{6}F^{16}	*Cahxk2Δ/Δ*	*Cahxk2ΔK^{6}F^{16} ::FRT/ Cahxk2ΔK^{6}F^{16} ::FRT*	This study

Table S2. Plasmids used in this study

Plasmids	Characteristics	Source or reference
pCaPC1c	Wild type genomic region of the *CaHXK2* gene cloned at the *Sph*1 *Xma*1 sites of pUC18	Laurian et al., 2019
pCaHXK2T^{15}A,	genomic region of the *CaHXK2* gene containing the T^{15}A mutation, cloned at the *Sph*1 *Xma*1 sites of pUC18	This study
pCaHXK2K^{6}F^{16}	genomic region of the *CaHXK2* gene containing the K^{6}F^{16}deleted region cloned at the *Sph*1 *Xma*1 sites of pUc18	This study
pCaHXK2D^{210}A	genomic region of the *CaHXK2* gene containing the D^{210}A mutation, cloned at the *Sph*1 *Xma*1 sites of pUC18	This study

Table S3. Primers used in this study

Name	Use	Sequence 5'->3'
p159 (F)	*CaHXK2* site-directed mutagenesis $D^{210}A$: change D at position 210 for A	GTTGATCAAC**GCA**ACCACAGGTACATTAGTTGCTTCTATG
p160 (R)	*CaHXK2* site-directed mutagenesis $D^{210}A$: change D at position 210 for A	TACCTGTGGT**TGC**GTTGATCAACGCAACAACATCAATTGG
p161 (F)	*CaHXK2* site-directed mutagenesis $T^{15}A$: change T at position 15 for A	GAAAGGGA**GCA**TTCACTGATGTTTCTCCTCAATTATTAGAAGC
p162 (R)	*CaHXK2* site-directed mutagenesis $T^{15}A$: change T at position 15 for A	CATCAGTGAA**TGC**TCCCTTTCTTTTTTGAGCTGGTTTTGG
p163 (F)	*CaHXK2* site-directed mutagenesis: deletion of the region between K at position 6 to F at position 16	CATCTCGGTCCAACTGATGTTTCTCCTCAATTATTAGAAGC
p164 (R)	*CaHXK2* site-directed mutagenesis: deletion of the region between K at position 7 to F at position 16	GAAACATCAGTTGGACCGAGATGCACCATTGTAAG
pHGT12 (F)	Amplification of *CaHGT12*	GGTACTCAAATTCTTCAAGCGTTC
pHGT12 (R)	Amplification of *CaHGT12*	GACGCCATAACAACACTACCG
pACT1 (F)	Amplification of *CaACT1*	ATTGTTTCCAACTGGGACGAT
pACT1 (R)	Amplification of *CaACT1*	GATGTTCTTCTGGAGCAACTCT
pICL1 (F)	Amplification of *CaICL1*	CTACTTTATCATTGGTGCCACTAACCC
pICL1 (R)	Amplification of *CaICL1*	AGACTCAATGGCAGCTAATTCGTTACC
pPCK1 (F)	Amplification of *CaPCK1*	GGTCCAAAAGTTGTCTCTTAATACCG
pPCK1 (R)	Amplification of *CaPCK1*	GCACCAGTAGATGAGATAGTAGTACC

Nucleotides in bold correspond to the changes inserted by site directed mutagenesis
